# The effect of a coach motivational micro-climate intervention in swimming: a mixed-method evaluation

**DOI:** 10.3389/fpsyg.2026.1771962

**Published:** 2026-03-31

**Authors:** Kylie Moulds, Shaun Abbott, Stephen Cobley

**Affiliations:** 1 Discipline of Exercise and Sport Science, Faculty of Medicine and Health, The University of Sydney, Sydney, NSW, Australia; 2 Tennis Australia, Melbourne, VIC, Australia; 3 School of Science, Technology and Health, York St John University, York, United Kingdom

**Keywords:** bioecological theory, coach education, coach motivational climate, dropout, youth sport

## Abstract

Theoretically informed by bio-ecological theory, research on coach-created motivational climates, and concerns regarding youth sport dropout in swimming, this study aimed to (1) develop and implement a motivational climate coach intervention for swimming coach development; and (2) evaluate intervention effects via swimmer and coach mixed-methods assessment. A quasi-experimental, repeated-measures, mixed-methods intervention was implemented over a 12-week period. Accredited Australian swimming coaches (*N* = 19) from 19 clubs participated, with coaches alternately allocated into intervention or control groups. To determine pre-post intervention effects, affiliated swimmers (*n* = 112 intervention; *n* = 97 control)—not informed of coach grouping—completed the Empowering and Disempowering Motivational Climate Questionnaire (EDMCQ-C) at two timepoints (pre- and post-intervention follow-up). Meanwhile, coaches self-evaluated their coaching behaviours using the EDMCQ at similar time points. Findings indicated that, relative to controls, swimmers connected with intervention coaches reported higher empowering and lower disempowering coaching behaviours post-intervention, after adjusting for baseline ratings, with medium effect sizes. A greater empowerment gain was apparent among swimmers who rated their coach lower at baseline. Contrastingly, no pre-post changes were apparent in coach self-ratings. Qualitative interviews with intervention coaches identified perceived intervention benefits (e.g., communication styles) and impact on coaching behaviours. Findings suggested that coach motivational climate training could benefit knowledge and behavioural strategies for improving the youth sport experience and identified future research recommendations. Whether improved coaching climates can translate into broader impact on youth sport outcomes remains uncertain.

## Introduction

Bronfenbrenner’s (1979) original Ecological Human Development Theory focuses on the nature of developmental outcomes resulting from behavioural interactions between individuals nested within broader social contexts. Bronfenbrenner (1986) argued behaviours could be understood by examining four surrounding systems interacting with the individual: the micro-, meso-, exo-, and macro-systems. Continuing (un-) stable relationships and interactions over time in these system types were proposed to bi-directionally influence the individual’s psychosocial functioning. Functional (e.g., positive psychological, social, and physical health) or dysfunctional development (e.g., sport dropout, psychological problems) outcomes likely occurred due to the nature of frequent, sustained social exchanges within developmental activities in micro-system contexts (e.g., sport and education).

Within youth sport, the coach–athlete relationship constitutes a salient micro-system in which coaching behaviours, interactional styles, and relational cues function as primary vehicles for conveying, interpreting, and internalising meaning by young athletes (Bronfenbrenner, 1979; Bronfenbrenner and Morris, 1998; Mageau and Vallerand, 2003). From this perspective, coaching behaviours are not merely instructional acts, but developmentally consequential social processes that shape athletes’ motivation, engagement, and psychosocial functioning over time (Smith et al., 2007; Keegan et al., 2014).

Complementing this ecological framing, motivational climate theory provides an empirically validated framework for specifying how coaches structure achievement environments and the psychological signals transmitted through their behaviours (Ames, 1992; Duda, 2013). Duda’s empowering climate framework (Duda, 2013) distinguishes between coaching behaviours that support autonomy, competence, and relatedness (empowering), and those characterised by control, ego-involvement, and pressure (disempowering; Appleton et al., 2016; Bartholomew et al., 2010). These dimensions offer a theoretical operationalisation of coach–athlete interactional quality within the micro-system (Duda and Appleton, 2016).

In the present study, bio-ecological theory and motivational climate theory are not treated as parallel explanatory frameworks, but as analytically complementary. Motivational climate constructs are positioned as operational indicators of proximal processes occurring within the coach–athlete micro-system (Bronfenbrenner and Morris, 1998; Duda, 2013). Bio-ecological theory attempts to explain these motivational processes within a developmental systems framework, highlighting their cumulative effects over time (Bronfenbrenner, 2005). This integration allows coach-created motivational climates to be examined not only as psychological environments, but as developmentally meaningful micro-system processes with potential implications for sustained sport engagement (Keegan et al., 2010; Moulds et al., 2022).

### Coach motivational climate interventions

Empowering coaching climates, as hypothesised to convey positive information and meaning, suggest that their promotion and stable implementation would be beneficial to youth sport. Indeed, previous intervention studies have been conducted, including the Promoting Adolescent Physical Activity (PAPA) project (Duda et al., 2013; Quested et al., 2013) alongside isolated studies in several areas of North America. The PAPA project investigated the potential of youth sport to promote children’s mental and emotional health, and engagement in physical activity. Within the project, coach education interventions aimed to increase ‘grassroots’ coaches’ understanding of how to support the experiences, motivation, and enjoyment of youth aged 10–14 years (The PAPA project, 2023). For example, with French and Norwegian youth grassroots soccer coaches (*N* = 18), a 6-h pre-season workshop, supplemented by workbook activities and online modules, introduced and examined the application of empowering motivational strategies in their coaching and behaviours. Focus groups conducted post-season with coaches revealed that the intervention was particularly helpful in changing coaching behaviours (more autonomy support, involvement, and structure) and implementing strategies to stimulate long-term enjoyment and participation behaviours (Larsen et al., 2015).

Similarly, in a United States community-based youth basketball program involving 37 coaches [*n* = 4 women, *n* = 33 men; *M*_age_ = 45.0, standard deviation (SD) = 6.17] and *N* = 216 junior players (*n* = 99 women, *n* = 117 men; *M*_age_ = 11.50, SD = 1.63), Smith et al. (2007) implemented the Mastery Approach to Coaching (MAC) intervention across a 12-week basketball season. The intervention involved training coaches to create a mastery-oriented motivational climate by emphasising effort, learning, and positive reinforcement. Players whose coaches completed the intervention perceived a more mastery-involving climate than those in the control group. Over time, athletes in the control condition showed significant increases in sport anxiety. In contrast, those in the intervention group did not, highlighting the protective effect of a mastery-oriented coaching climate.

While coach-created empowering motivational climate and associated strategies have been identified to facilitate favourable psychological outcomes (i.e., enjoyment, task cohesion, and anxiety; Appleton et al., 2016) in youth sport participant contexts, the idea of developing empowering instructional strategies and behaviours within coaching practice has been less examined. Improving coaching motivational climates through a coach development intervention could improve participants’ experience, cognitive–emotional perceptions, and/or behavioural outcomes such as sustained engagement or dropout prevention. However, no study—to date—has specifically attempted to improve coach-created motivational climates to address youth sport dropout. Building on previous research (Moulds et al., 2020; Moulds et al., 2022) demonstrating dropout rates among Australian youth swimmers and the influence of coaching behaviours on swimmer motivation, the present study further explores the environment that shapes sustained participation.

### Study purposes

Grounded in concerns regarding youth sport dropout and informed by bio-ecological and motivational climate theory, the present study examined whether a short-course coach development intervention could modify coach-created motivational climates within competitive youth swimming. While dropout behaviour was not directly measured, motivational climate characteristics targeted by the intervention are theoretically and empirically linked to athlete engagement, enjoyment, and sustained participation.

Specifically, the study aimed to:

(1) Develop and implement a theory-informed coach development intervention designed to enhance empowering and reduce disempowering coaching behaviours and
(2) Evaluate intervention effects using swimmer-reported motivational climate perceptions, coach self-reports, and qualitative interviews to explore perceived behavioural change mechanisms.

The term *motivational micro-climate* is used in this study to describe the athlete’s lived and perceptual experience of the coach-created motivational climate within the immediate coach–athlete relationship. While grounded in established empowering–disempowering climate constructs, the micro-climate concept emphasises the ecological immediacy of these interactions—that is, how motivational signals are experienced moment-to-moment within training and competition settings. Accordingly, motivational micro-climate is not proposed as a novel construct, but as an ecological refinement that situates motivational climate perceptions within the microsystem level of development.

## Methods

### Participants

Following University human research ethics approval (Approval No: 2022/442) and participant consent, the initial cohort of coach participants (*N* = 22) consisted of swimming coaches from New South Wales, Australia. However, three participants withdrew following initial survey completion and prior to intervention. Reasons provided were associated with due parallel work commitments and a preference not to have their swimmers rate their coach’s motivational micro-climate. Final coach participants were *N* = 19 (*n* = 6 women; *n* = 13 men). Correspondingly, *N* = 208 swimmers (*n* = 97 women; *n* = 111 men) who were affiliated with their respective coaches at the time of study completion agreed to participate. Participating coaches, swimmers, and clubs resided across urban and rural New South Wales, Australia.

*Recruitment and inclusion/exclusion criteria.* Coaching involvement was promoted via Swimming New South Wales^1^ (SNSW) internal and external communication channels (e.g., email and social media). Interested coaches were screened for eligibility and included if they were accredited with the Australian Swimming Coaches and Teachers Association (ASCTA) at a development or advanced level and had been coaching at their current club for ≥12 months. Following coach enrolment, SNSW facilitated contact with affiliated swimmers via club representatives, who explained study procedures and invited voluntary participation. Swimmers were eligible if aged 10–18 years, registered as SNSW competitive swimmers, and had worked with their coach for ≥12 months. Swimmers remained unaware of the coach group allocation to minimise evaluation bias (Schulz et al., 1995), and were advised not to discuss study involvement during data collection. As discussed in the study by McLaren et al. (2015), a 1:10 coach–swimmer sampling ratio was targeted, with swimmer ratings prioritised due to their independence from coach treatment.

### Research design and procedures

**Figure 1:**
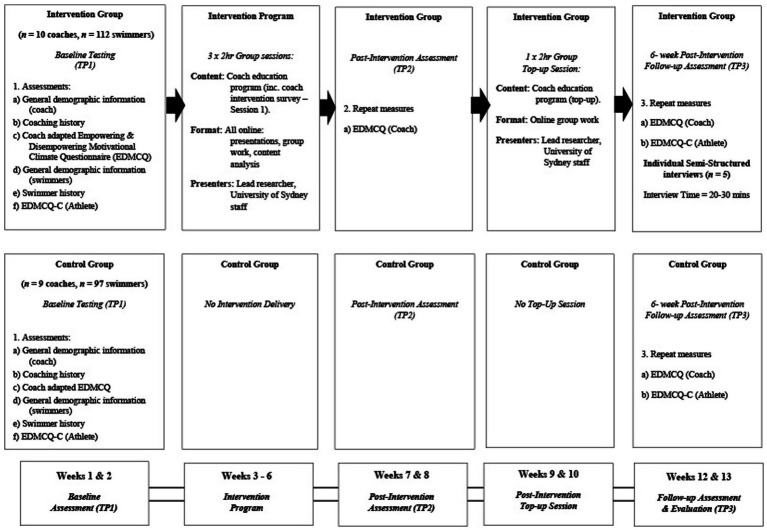
An overview of the coach-created micro-climate intervention program design and data collection stages.

A quasi-experimental, mixed-method design was implemented to determine intervention effects (see Figure 1). Mixed-method approaches are well-suited to applied research, enabling triangulation between quantitative and qualitative data (Kegelaers et al., 2019; Morse and Niehaus, 2009). Following enrolment, coaches were alternately assigned to the intervention or control group according to sign-up order. Swimmers were not made unaware of the coach group allocation, and coaches were asked not to disclose the grouping. For both swimmers and coaches in the intervention and control groups, consistent questionnaire completion occurred pre-intervention (TP1) and at the 6-week follow-up (TP3). Intervention coaches completed an additional round of questionnaire completion at TP2.

#### Swimmer assessment

Providing independent assessment, at TP1 and TP3, all swimmers—blinded to coach group allocation—completed the Empowering and Disempowering Motivational Climate Questionnaire (EDMCQ-C) (Appleton et al., 2016) via Research Electronic Data Capture (REDCap). REDCap is a secure, web-based software platform that supports data capture (Harris et al., 2009). As we expected minimal/no immediate coach behaviours change, TP2 expected minimal/no change; time was permitted for swimmer recognition, accounting for data collection at TP3 (6 weeks post-intervention). Swimmers aged <15 years were encouraged to complete the EDMCQ-C with parental support. Completion required approximately 10–15 min, and all data remained confidential.

The 34-item EDMCQ-C assesses coach-created motivational climate characteristics across two higher order *Empowering* and *Disempowering* dimensions, each containing 17 items. The *Empowering* dimension contains three sub-components: *autonomy support* (5 items), *social support* (3 items), and *task-involving climate* (9 items); while the *Disempowering* dimension contains two sub-components: *ego-involvement* (7 items) and *controlling coach behaviour* (10 items). Items reflected typical coach behaviours and interactions during training and competition over the last year and were rated on a five-point Likert scale (1 = *strongly disagree*, 5 = *strongly agree*). The EDMCQ-C has demonstrated validity and reliability in youth sport (Appleton et al., 2016). Internal consistency in swimmer ratings ranged between Cronbach’s *α* = 0.83–0.87, suggesting internal consistency (Cohen, 2013).

#### Coach assessment

At TP1, TP2, and TP3, intervention coaches completed an online REDCap version of the EDMCQ (Appleton et al., 2016) adapted for swimming, while control coaches completed it only at TP1 and TP3. The 34-item EDMCQ assessed similar high-dimensional *Empowering* and *Disempowering* constructs, along with the five lower order sub-components, but from a coach self-reporting standpoint. The EDMCQ asked coaches about their coaching behaviours and interactional styles they had deployed over the last year within swimming training and competition contexts. Item responses were rated on a five-point Likert scale (1 = *strongly disagree*, 5 = *strongly agree*). Cronbach’s α for swimmer and coach responses ranged between 0.79 and 0.90, suggesting a high level of internal consistency (Cohen, 2013).

#### Coach semi-structured interviews

To provide experiential insight into intervention effects, semi-structured interviews were conducted at TP3. Interviews exploring coaches’ motives for participation, perceived benefits and impacts of interventions, and recommendations for future improvements (Creswell, 2015; Tenny et al., 2022). Interview schedules were informed by previous research (Cupples et al., 2021; Smith et al., 2007) and piloted with two similarly qualified swimming coaches (see Appendix A). Open-ended questions were used to elicit coaches’ perspectives and experiences. Five intervention coaches (3 men and 2 women) participated, with interviews lasting an average of 25 min, and were subsequently transcribed verbatim.

### Intervention content

Informed by theoretical frameworks (Bronfenbrenner & Morris, 2008), prior empirical studies (Duda, 2013; Duda and Appleton, 2016), as well as interventions in basketball, soccer and rugby league (Cupples et al., 2021; McLaren et al., 2015; Smith et al., 2007), an online short-course intervention was developed (see Table 1). The intervention consisted of three 2-h, group-based, interactive, online educational training sessions, delivered over consecutive weeks. Three weeks after TP2, an additional 2-h session was conducted. The three main sessions were organised into the following six components: the first two sessions (covering components 1–4) focused on developing coaches’ knowledge and understanding of motivational and motivational–climate concepts, and the final session (components 5–6) introduced Bronfenbrenner’s bio-ecological model (Bronfenbrenner, 2005; Bronfenbrenner and Morris, 1998). Then, emphasis was placed on how to develop and create more preferable motivational climates within a swimming club environment. Sessions included presentation content, interactive and individual task activities, and small group discussions. Intervention content was delivered by the lead author with assistance from fellow authors. Control group coaches were given the opportunity to receive the intervention following study completion.

**Table 1:** An overview of the coach micro-climate intervention program content.

Session	Topic	Session content	References	Session type	Presenter
1	Coaching climate intervention program overview. Defining motivation and motivational coaching climates (empowering/disempowering)	Coach climate questionnaire – setting the scene		LF	University of Sydney staff (SC) Lead author
		Outline the purpose of the program – Study 1–3 findings	Moulds et al. (2020) and Moulds et al. (2022)	LF	Lead author
		Raising awareness of the dropout challenges within NSW youth swimming	Moulds et al. (2020) and Moulds et al. (2022)	LF	
		Defining motivation and motivational coaching climates	Deci and Ryan (1985, 2000) and Appleton et al. (2016)	SG	
		Introduction to empowering and disempowering climates	Duda (2013), Appleton and Duda (2016), Empowering Coaching (2019), and Appleton et al. (2016)	GD	
2	Building blocks for positive outcomes in sport	Building blocks for positive coaching	Empowering Coaching (2019)	GD	Lead author
		Autonomy, belonging, and competence in swimming – ABCs of coaching climates	UK Coaching (2019), and Appleton et al. (2016)	LF	
			Deci and Ryan (2000, 2005), Ames (1992), and Nicholls (1989)	GD	
		Case study: PAPA project – how to identify ABCs	Smith et al. (2007) and Appleton et al. (2016)	SG	
3	Creating empowering environments – Coaching strategies	Creating empowering coaching climates	Duda (2013), Appleton and Duda (2016),Empowering Coaching (2019), and Appleton et al. (2016)	LF	Lead author
		Discussion: ‘What does an empowering coaching environment look like in youth swimming?’		GD	
		Empowering climate dimensions	Deci and Ryan (1985, 2000), Ames (1992), Nicholls (1989), and Appleton et al. (2016)	SG	
		Evaluation group discussion: barriers to an empowering climate	Duda (2013), Empowering Coaching (2019), and UK Coaching (2019)	GD and CA	
4	Creating empowering environments – summary	Empowering coaching climates in practice		LF	Lead author
		Evaluation group discussion: examples from own coaching sessions	Empowering Coaching (2019)	GD and CA	
5	Impact of other socio-environmental factors in youth sport	Introduction to Bronfenbrenner’s bio-ecological model and how this applies to youth sport dropout/continuation	Bronfenbrenner (1979, 2005)	LF	Lead author
		Strategies: How to apply the Process–Person–Context–Time (PPCT) model to help understand youth swimmers.	Bronfenbrenner and Morris (1998)	LF and GD	
		Case studies: link between the PPCT model and empowering coaching behaviours.	Bronfenbrenner and Morris (1998) and Empowering Coaching (2019)	CA	
6	Long-term application of empowering climates in individual coaching scenarios	Developing holistic motivational action plans		LF, GD, and CA	University of Sydney staff (SC) Lead author
		Discussion: common barriers to continued participation in swimming		CA	
		Intervention program summary and next steps Program evaluation		CA, GD	

Sessions 2–6 started with a review of the previous session and a brief discussion. Opportunities for further questions were provided post-session. Session delivery – lecture format (LF), small group discussion and presenting (SG), group discussion (GD), and content analysis (C).

### Data analysis

#### Swimmer assessment

Swimmer EDMCQ-C data were screened for missing or invalid responses, with no outliers identified via boxplot inspection. Composite empowering and disempowering scores were calculated at baseline (TP1) and follow-up (TP3). Given that swimmers were nested within coaches, and the intervention was delivered at the coach level, intervention effects were examined using separate hierarchical mixed-effects ANCOVAs for empowering and disempowering climates. TP3 group differences were tested while adjusting for baseline scores as covariates, with coach specified as a random intercept to account for clustering. Intraclass correlation coefficients (ICCs) were calculated to quantify variance attributable to coach affiliation. Model assumptions, including baseline equivalence, homogeneity of regression slopes, normality, residual independence, and homoscedasticity, were assessed and met. Statistical significance was set at *p* < 0.05. Effect sizes were reported as standardised adjusted differences (*d*_DiD_), calculated as the between-group TP3 difference divided by the pooled baseline standard deviation and interpreted using Cohen’s (1988) criteria. To verify primary findings, Group × Time interaction analysis of variance (ANOVA) tests were conducted, with assumptions checked and effect sizes reported as partial eta squared (η^2^).

#### Coach self-assessment

Given the limited sample size of coaches and the differential data structure between groups, coach self-report ratings were analysed exploratorily using ANOVA. Two mixed “Group (intervention vs. control) × Time (TP1 vs. TP3)” ANOVA tests were conducted on self-rated empowering and disempowering scores. Similar assumption checks, alpha, and effect size criteria as per swimmer analysis were completed. All statistical analyses were conducted in Statistical Package for the Social Sciences (SPSS) version 25.0 (Chicago, IL, United States).

#### Interviews

Transcripts were analysed using reflexive thematic analysis, adhering to Braun and Clarke’s (2006, 2019) six-step framework. Familiarisation involved repeated reading of transcripts and one-page summaries, which were returned to coaches for accuracy checking (Korstjens and Moser, 2018). Initial themes were developed deductively, with raw data coded and organised in QSR NVivo software (version 1.1.1; Fereday and Muir-Cochrane, 2006; Strauss and Corbin, 1998). Data transcripts were coded and organised into themes, with frequency counts (FCs) and references used to examine emerging themes. To enhance the trustworthiness of data interpretation, several strategies were employed (Lincoln and Guba, 1985; Korstjens and Moser, 2018). During interviews, coaches were prompted to provide examples, with follow-up questions used to clarify meaning. Triangulation was achieved through interview transcripts and recordings (Sim and Sharp, 1998), as well as the research team’s analytic review. Researchers acted as critical friends to ensure analytic rigour and alignment with study aims and context (Tracy, 2010). Credibility was ensured through the interviewer (KM) undertaking verbatim transcription, analysis, and reflexive discussion with the research team to support theme refinement and inform the development of the summary table (see Table 2).

**Table 2:** A reflexive thematic analysis summary of coach semi-structured interviews evaluating the coach micro-climate intervention program.

Theme	FC (R)	Codes	FC (R)	Description	Exemplar quote
Participation motives	5 (16)	Networking	4 (7)	Participants sought information from other coaches. A dynamic and evolving informal coaching network, often described as a dynamic social network (Mallett et al., 2009).	‘In the past, it’s valuable being able to discuss ideas with other coaches.’ Coach_5
		Coach education	2 (4)	Professional knowledge is conceptualised by a coach’s ability to know what (declarative) and how (procedural) to operate in the coaching environment (Abraham et al., 2006).	‘I did not have any preconceived idea of what I was coming in for, my motivation from the program was to learn [sport psychology] and contribute.’ Coach_4
		Coach development	3 (3)	Interpersonal, bi-directional relationships that are present in the coaching environment and intrapersonal knowledge of oneself (Côté and Gilbert, 2009).	‘Really, my main motivation for participating in the program was probably my situation with a swimmer because it’s really challenged me.’ Coach_5
		Share new information	1 (2)	Head coach learning new information to share it with their own coaching staff.	‘I was motivated to attend so I can share any new information with my coaching staff.’ Coach_2
Participation benefits	5 (52)	Communication styles	5 (15)	The process of sending and receiving information between the coach and the athlete.	‘Communication strategies were a big eye-opener for me in terms of, athletes are not all the same. This might work for me or for a certain individual, but it might not work for the other athletes.’ Coach_4
		Coach development	2 (13)		‘Conversations with other coaches during the program have given me a lot more conscious thought on what I’m doing and why I’m doing it.’ Coach_2
		Reassurance	4 (10)	The information covered during the program served as a reminder or reinforcement of prior knowledge and current coaching behaviours (coaching practice).	‘With many years of experience, I had heard of some key concepts before but maybe I had not used a strategy for a long time, so I was able to come back to it.’ Coach_3
		Coach education	4 (10)		‘I felt like there was new information which encouraged me to adapt my coaching practice and introduce some new concepts into training.’ Coach_5
		Share new information	2 (4)		‘The program really helped me to have more conversations with my assistant coaches.’ Coach_1
Intervention impact – Bronfenbrenner’s bio-ecological model	5 (16)	Coach–parent interaction patterns	3 (8)	Coach–parent interactions that can influence athlete development meso-system of Bronfenbrenner’s bio-ecological model (1979). The importance of viewing parents as assets, coaches working or collaborating with parents (Harwood, 2011; Harwood and Knight, 2009), rather than as problems to be fixed (Bruner et al., 2020).	‘It’s important to encourage the parent/s and help educate them, to work with them rather than against.’ Coach_4
		Interrelations of micro–macro systems	3 (8)	Nested systems consider the interrelations between different layers of Bronfenbrenner’s model (1979) when examining athlete development.	‘Every swimmer comes through the door with different issues going on each day, so from my perspective, I need to manage this.’ Coach_5
Intervention impact – empowering coach framework	4 (18)	Autonomy	2 (4)	Coach-created motivational climate that can optimise the what, why, and how of child and adolescent athletes’ continued participation (Duda, 2013). Coach-created environment that facilitates relative choices and options for child and adolescent athletes.	‘It’s important for the coach and the group to understand empowerment, for the swimmers to feel psychologically safe enough to tell the coach how they are feeling or how something might be affecting them.’ Coach_5
		Belonging	2 (8)	Coach-created motivational climate that facilitates a sense of connection/belonging for the athlete.	**‘**From a child’s perspective, little things such as a team chant go a long way to have them feel a sense of belonging within each group.’ Coach_3
		Competence	3 (4)	Coach-created motivational climate which is more task-orientated with a clear focus on the process rather than the outcome.	‘When a swimmer got a personal best (PB) in training we celebrated with that swimmer, so they felt valued.’ Coach_2
Future preferences	5 (11)	Delivery	2 (2)	Program delivery mode.	‘The delivery mode could be improved, to be honest, I prefer to meet face to face.’ Coach_3
		Timing	4 (8)	The duration of each session (h) and start/finish time	‘Being coaches we are up early in the morning and the timing of the sessions could be better, to be scheduled directly after early morning training sessions.’ Coach_4
		Theory to practice learning	1 (1)	Commencing each session with theory and relaying this information directly into a practical environment.	‘It would be good to spend time at the pool and then afterwards have all of our club coaches sit down and go through a program like that together.’ Coach_1

Frequency count (FC), No. of coaches mentioned the theme/code; Reference (R), the total number of the identified themes/codes.

## Results

### Intervention and control group descriptives

The intervention group comprised 10 coaches (*M*_age_ = 36.44, SD = 10.86) with a mean of 9.7 years (SD = 7.75) of coaching experience. In the previous year, they averaged 27.5 h/week (SD = 14.00) coaching with ~50.5 swimmers (SD = 25.14). A total of 112 swimmers (*M*_age_ = 13.29, SD = 1.55) were affiliated with intervention coaches, having trained with respective coaches for 2.26 years on average (SD = 1.38). Swimmers reported competing in 9.94 meets/year (SD = 5.35) and training for 10.1 h/week (SD = 5.67). Coach intervention adherence averaged 97% (range = 90.4–100%); three coaches missed one session but completed content via recording. All swimmers completed ratings at TP1 and TP3.

The control group included nine coaches (*M*_age_ = 39.12, SD = 13.50) with 13.72 years of experience (SD = 7.75). In the previous year, they averaged 30.65 h/week (SD = 15.71) of coaching with ~77.7 swimmers (SD = 36.75). Ninety-seven swimmers (*M*_age_ = 13.23, SD = 1.55) were affiliated, training with control coaches for an average of 2.64 years (SD = 1.46). Swimmers reported competing in 9.86 meets/year (SD = 5.25) and completed *M* = 10.86 h/week of training (SD = 4.01). All control coaches and swimmers completed study requirements.

### Swimmer assessment

Mixed-effects ANCOVAs identified significant differences between intervention and control group swimmer ratings for coach motivational climate evaluations at TP3 [Group (Empowering): *b =* 0.03, standard error (SE) = 0.01, *t* = 3.84, *p* < 0.01, *F* (1, 18.51) = 14.75; Group (Disempowering): *b =* −0.03, SE = 0.01, *t* = −2.69, *p* = 0.02, *F* (1, 16.09) = 7.26], after controlling for baseline ratings (Empowering baseline: *b =* 0.95, SE = 0.01, *t* = 107.92, *p* < 0.01, *F* (1, 199.67) = 11,647.39; Disempowering baseline: *b =* 0.95, SE = 0.01, *t* = 90.97, *p* < 0.01, *F* (1, 204.47) = 8,275.17). Medium group (intervention vs. control) difference effect sizes were apparent [empowering *d*_DiD_ = 0.08, 95% confidence interval (CI) = 0.03–0.12] disempowering (*d*_DiD_ = −0.08, 95% CI = −0.15 to −0.02). Regarding empowerment ratings, the linear relationship between Group at TP3 was influenced by variations in baseline (TP1) swimmer ratings, indicating an interaction (*b* = −0.07, SE = 0.02, *t* = −3.89, *p* < 0.01). Specifically, the intervention was more positively effective amongst swimmers who rated their coaches lower on empowerment at baseline. No interaction was apparent for disempowerment ratings. The inclusion of a coach random effect identified approximately 3% (empowering) and 13% (disempowering) of variation in swimmers’ scores as attributable to coach affiliation at TP3 (*τ*_00_ < 0.01, ICC = 0.03 and 0.13). The results imply that differences in coach motivational climate were marginally influential. To evaluate the statistical power of the modelling approach, simulation-based power analyses were conducted using the simr package in RStudio (Green and MacLeod, 2016). When the standardised target interaction coefficient was simulated at 0.30 SD (a medium effect size of 0.30), power to detect the Time × Group interaction at *α* = 0.05 was 100% (95% CI = 99.63–100.00). Thus, the study design and sample were sufficiently powered to reliably detect moderate effects.

**Figure 2:**
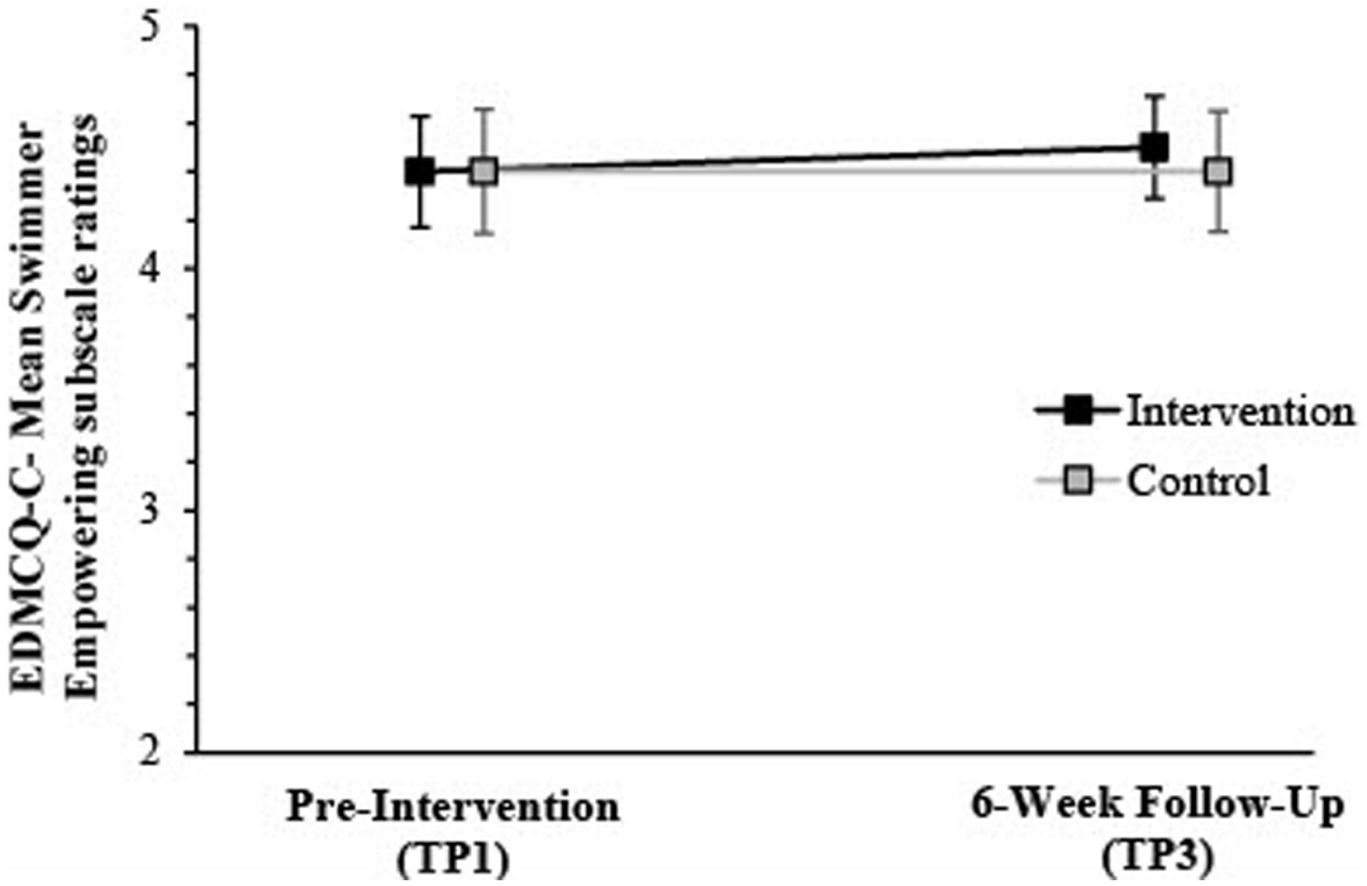
Mean & 95% CIs for swimmer ratings on EDMCQ-C empowering subscales at pre-intervention (TP1) & 6-week post-intervention follow-up (TP3) according to intervention and control group.

**Figure 3:**
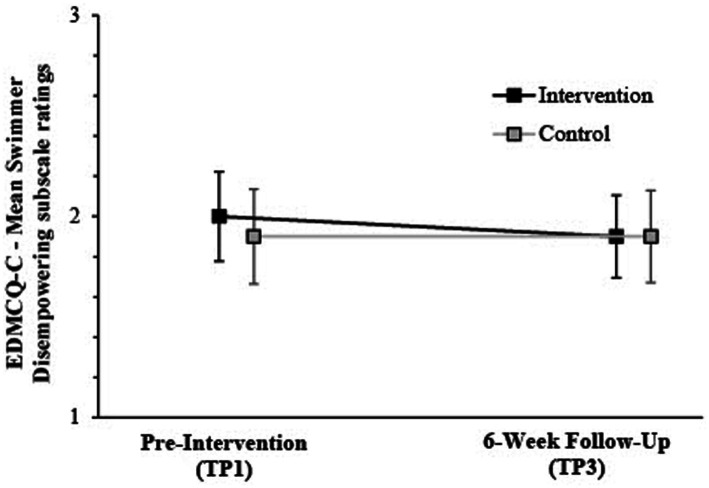
Mean & 95% CIs for swimmer ratings on EDMCQ-C disempowering subscales at pre-intervention (TP1) & 6-week post-intervention follow-up (TP3) according to intervention and control group.

Follow-up ANOVA tests identified a significant Group × Time interaction for empowering scores, with a medium effect size [*F* (1,206) = 15.87; *p* < 0.001; Wilks’ *Λ* = 0.98; partial *η^2^* = 0.07]. A similar interaction was apparent for disempowerment, medium effect size [*F* (1,206) = 22.32; *p* < 0.001; Wilks’ *Λ* = 0.99; partial *η^2^* = 0.10]. Swimmers of intervention coaches increased empowering ratings from TP1-TP3 (*M*diff = 0.04), with disempowering ratings decreasing (*M*_diff_ = −0.004). Meanwhile, control-associated swimmers reported no significant change. Irrespective of group, main time effects were apparent [Empowering = *F* (1,206) = 36.17, *p* = 0.001, partial *η^2^* = 0.15; Disempowering = *F* (1,206) = 25.89, *p* = 0.001, partial *η^2^* = 0.11] (see Figures 2, 3).

### Coach self-assessment

The exploratory Group × Time ANOVA tests did not identify interactions [Empowering = *F* (1,16) = 2.65, *p* = 0.12; Disempowering = *F* (1,16) = 1.05, *p* = 0.31], nor a main Group [Empowering = *F* (1,16) = 2.65, *p* = 0.12; Disempowering = *F* (1,16) = 3.64, *p* = 0.08] or Time [Empowering = *F* (1,16) = 2.91, *p* = 0.10; Disempowering = *F* (1,16) = 1.40, *p* = 0.25] effect. Only descriptive trivial intervention group changes were reported (TP1-TP3 empowering = *M*diff = +0.03; disempowering *M*_diff_ = −0.03).

### Qualitative coach interviews

Reflexive thematic analysis identified five themes relating to intervention participation and perceived effectiveness: *participation motives*; *participation benefits*; *intervention impact* (Bronfenbrenner’s bio-ecological model); *intervention impact* (empowering coaching); and *future preferences* (see Table 2). Themes comprised component names, with frequency counts (FCs) indicating the number of coaches who discussed the theme, and references (R) indicating the total number of transcript mentions.

During interviews, coaches’ primary motivations for intervention engagement (FC = 5, R = 16) were associated with opportunities for peer *networking* (FC = 4, R = 7), *development* (FC = 3, R = 3), *education* (FC = 2, R = 4), and *information exchange*. Connected to underlying motives, five themes of benefit in intervention were described, eliciting the greatest number of transcript references (FC = 5, R = 52). Benefits to intervention coaches centred around understanding the impact of *communication styles*, and how utilising and developing alternative styles could facilitate athletes’ responses (FC = 5, R = 15). Sample coaches perceived that intervention content, as well as interactions with other coaches, had generated reflective thought on personal practices; helped identify strategy and practice modification (*coach development*; *coach education*); and helped exchange information and ideas, *sharing new information*. For some, the intervention content provided *reassurance* about knowledge as well as a clearer framework for coaching behaviours already adopted.

“*Talking with other coaches in our groups…, has given me a lot more conscious thought on what I’m doing and why I’m doing it.*” (Coach 2)

When discussing specifically how the intervention had an impact, coaches spoke of better consideration of inter-relationships of those involved in the athlete micro-system; a theme aligned with Bronfenbrenner’s bio-ecological model (FC = 5, R = 16). Coaches identified how the intervention informed and reinforced the value of coach-parent interaction patterns, emphasising the merits in collaboration alongside swimmers: “*I want to communicate with the parents rather than fight against them, it’s important to be on the same page*:” (Coach 4); “*Parents can help us to have a better understanding of what’s being reinforced at home*” (Coach 1). Improved micro–macro inter-relations were also identified (FC 3, R = 8), with coaches seeing the value and reciprocal benefit from “*getting to know swimmers on more of a personal level; getting to know their lives helped or hindered their swimming.*” (Coach 2). In alignment, Coach 5 described how intervention content helped recognise how “*every athlete walks into the room with different issues each day (school, home, peer group), so understanding how this might affect them and have an impact on their training is important*.” Thus, recognising and understanding swimmer behavioural variations affecting engagement, and being able to deploy more helpful, impactful coach interactional behaviours at that time point was deemed important.

Viewed with an empowering coaching framework lens, intervention impact (FC = 4, R = 18) aligned with three lower order components: *Belonging*, *Autonomy*, and *Competence*. Across interview responses, coaches described more explicit attempts to implement empowering behavioural strategies within their practice, including strategies to promote athlete responsibility (e.g., leadership in training activities), group cohesion (e.g., fostering shared team rituals), and positive reinforcement (e.g., recognising effort and personal best achievement to support competence). Coaches also reported using swimmer self-evaluation strategies to encourage reflection and self-controlled development. Collectively, these examples supported the assertion that empowering principles from intervention can be translated into practice. That said, discussion of disempowering behaviours and their potential reduction was not mentioned in responses.

Related to *future preferences* for intervention delivery, all coaches provided recommendations (FC = 5, R = 11). As coaches balanced early and late-day swimming training and work schedules, *Timing* considerations were emphasised. Coach education, thus, had to be considerate of parallel engagement demands. In *Delivery*, recommendations were towards shorter session durations (compared to 2-h intervention content), with suggestions of utilising a mixture of pre-recorded and face-to-face components. A better connection between outlined *Theory–practice* was emphasised, with a recommendation to implement the theoretical content immediately afterward in ‘poolside’ practice to maximise application.

## Discussion

This study examined the effects of coach development intervention in the context of SNSW youth swimming. Integrating Bronfenbrenner’s bio-ecological micro-system lens with Duda’s empowering–disempowering framework, the study targeted coaches’ interactional behaviours as a strategy to improve athlete experiences and participation engagement. Quantitatively via independent EDMCQ-C reporting and analysis of covariance (ANCOVA) analyses, swimmers affiliated with intervention coaches reported higher empowering and lower disempowering coach motivational climate ratings at 6-week follow-up relative to controls, with medium effect size group differences. Supplementary ANOVA tests corroborated findings, with identification of group-level interaction effects. Importantly, swimmers’ multiple independent ratings, obtained under instructed blinding, strengthen inferences of intervention behavioural transition effects, with swimmers recognising shifts in coach social-motivational tone within normative training environments. In contrast, coach self-ratings showed no pre-post change vs. controls. When comparing findings with similar prior literature examining coach motivational micro-climate interventions (Duda et al., 2013; McLaren et al., 2015; Smith et al., 2007), findings generally align, with coach climate modification positively affecting psychosocial outcomes (e.g., enjoyment, perceptions of group cohesion, and trait anxiety; Birr et al., 2023).

An important methodological consideration concerns the temporal alignment between the intervention duration and the EDMCQ-C measurement frame. The EDMCQ-C asks athletes to evaluate coaching behaviours over the preceding year, whereas the intervention spanned 12 weeks. This discrepancy may attenuate sensitivity to short-term behavioural change, as recent coaching behaviours are embedded within longer relational histories. From this perspective, the detection of statistically significant changes in swimmers’ perceptions is theoretically meaningful, suggesting that recent shifts in coaching behaviour were sufficiently salient to influence athletes’ global evaluations of their motivational environment. Nonetheless, future intervention studies would benefit from incorporating temporally sensitive or event-contingent measures to capture short-term behavioural change more precisely.

While positive inferences can be made, alternative explanations are also possible.

Due to being knowingly observed, intervention coaches may have increased behaviour responding (i.e., Hawthorne reactivity; Roethlisberger and Dickson, 1939; Sommer, 1968) to be motivated to demonstrate competence or conformity in a socially evaluative situation, rather than respond directly to the intervention content *per se*. Separately, (in-)advertent study involvement cross-contamination may have occurred (Magill et al., 2019; Stamp et al., 2021). Given the coach–swimmer power differentials, study involvement contamination could have affected swimmer rating independence. Still, as confidentiality was reinforced throughout, with swimmer involvement independently managed, this was considered less problematic. However, the discrepancy between swimmer and coach findings may suggest that self- or social-evaluation bias occurred (Hogan et al., 1997). Irrespective of group allocation, coaches’ baseline empowerment and disempowerment ratings were, respectively, high and low, which may have corresponded with ceiling or floor effects (Everitt and Skrondal, 2010), in which the intervention was unable to produce discernible effects (see, e.g., Girard et al., 2021). Relatedly, following baseline rating bias, intervention content may have served to actually recalibrate intervention coaches’ perceptions, realigning to more accurate, conservative ratings (i.e., response-shift bias—Howard, 1980). This may also account for the no changes in coach ratings. In contrast, a recruitment bias (Hamill et al., 1980) perspective would suggest that coaches’ skewed ratings were accurate. With coaches reporting extensive swimming coaching experience and stable club involvement, the sample may have genuinely been competent in implementing empowerment-aligned behaviours. Notwithstanding, qualitative interviews did triangulate with swimmer quantitative findings.

The intervention was designed to influence coaching behaviour through several complementary mechanisms. First, educational content was aimed at enhancing coaches’ cognitive awareness of how specific interactional behaviours convey motivational meaning to athletes. Second, structured reflection and peer discussion were intended to prompt recalibration of habitual communication styles. Third, applied tasks encouraged intentional enactment of autonomy-supportive, task-involving, and socially supportive strategies within training environments. Qualitative findings provide insight into these mechanisms, with coaches describing increased awareness of communication choices, greater intentionality in athlete interactions, and the adoption of concrete, empowering strategies, such as athlete leadership opportunities, effort-based reinforcement, and relational engagement. While direct behavioural observation was not undertaken, the convergence between swimmer-reported climate change and coach-described practice adjustments strengthens confidence in the proposed mechanism-to-outcome linkage.

In the intervention group, sample interviews with coaches described the impact of the intervention program, identified perceived benefits, and how they intentionally attempt to implement behavioural changes consistent with empowering coaching. Behaviours were centred around providing greater autonomy support (e.g., leadership opportunities), enhanced belonging (e.g., team rituals), and competence-supportive reinforcement (e.g., recognition of effort, personal bests). Coaches highlighted raised awareness in communication styles, internal reflective practice, and peer learning/networking opportunities as catalysts for transfer. Together, these accounts explained how coaches translated motivational climate recommendations into specific behavioural strategies. While interviewees did not explicitly emphasise reducing disempowering behaviours, swimmer data suggested a concomitant reduction, pointing towards either implicit de-emphasis of controlling/ego-involving practices or swimmer heightened sensitivity to empowering behavioural cues.

The mixed-method design enabled integration of quantitative outcome patterns with qualitative explanatory insight. Swimmer-reported increases in empowering and decreases in disempowering climate perceptions were elucidated by coaches’ accounts of altered communication practices, increased relational awareness, and intentional use of strategies. These qualitative findings extend the quantitative results by clarifying how coach development translated into observable changes in interactions within training contexts.

Rather than functioning solely as confirmatory support, qualitative data-informed interpretation of intervention mechanisms, contextual influences, and perceived transfer processes. This joint interpretation strengthens confidence in the intervention’s capacity to modify coach-created motivational micro-climates within a relatively short timeframe.

### Limitations

This study involved a self-selected coach sample in a specific sport context, with non-random allocation, potentially constraining generalisability. While intervention promotion was underway, only a limited number of swimming coaches volunteered. Anecdotal reasons for low uptake, provided by several sources, resonated with a pre-existing competitive coaching culture, the relative importance of intervention content to performance success, organisational or professional development recognition, and perceived fear of negative evaluation. Future studies should aim to recruit larger, more diverse cohorts to better reflect the broader coach population. Although the study’s finding of efficacy was derived from the large sample of independent swimmer ratings, ratings could have been affected by artificial coach responding from being observed and information contamination; while self-presentation, ceiling/floor effects, and response-shift bias could possibly influence coach ratings. On these grounds, recommendations to include behavioural verification (observation/video), extend tracking longevity, and to determine whether climate shifts translate into longitudinal reduction in youth dropout—key motivation for the present study.

### Implications

Study findings suggest that a theory-informed, short-course, coach development intervention can translate into athlete-recognised coaching micro-climate interactions and behavioural changes. If accurate and able to positively impact youth athlete’s experiences and engagement, sporting governing bodies, localised organisations, and their athlete members could benefit from implementing integrated coach development targeting local coaches. Paired with periodic micro-climate reviews to build coaches’ self-awareness and realignment of youth sport system values towards participation and development, this could assist youth coaching practice and culture change. If intervention content is delivered, modification towards shorter-duration sessions, delivered synchronously and *in situ* (theory application) with training and competition is recommended. Similarly, facilitating peer interaction, opportunities for knowledge and strategy ideas exchange, and applied implementation should be considered. To improve the finding of efficacy, future research should consider researcher observation and/or video recording to validate coach behaviour change, and/or incorporate extended follow-up to determine the intervention’s longevity on climate ratings and athlete outcomes (e.g., maintained engagement vs. dropout).

## Conclusion

In an Australian swimming context, a theory-informed, short-course, coach intervention produced detectable improvements in coach-created micro-climates within a 12-week timeframe. Relative to non-changing controls, swimmers affiliated with intervention coaches perceived increased empowering interactions and behaviours, while disempowering interactions and behaviours concomitantly reduced. Findings were triangulated through qualitative reports, in which coaches described the impact of interventions and their attempts to implement empowering behaviours in practice. Findings suggest that such intervention content can translate into observable changes in coach behaviour, recognisable by athletes. Such a change could be valuable to youth sport experiences, helping address concerns such as dropout.

## Data Availability

The raw data supporting the conclusions of this article will be made available by the authors, without undue reservation.

## Footnotes

1 Swimming New South Wales (SNSW), a state affiliate of Swimming Australia (SAL), implements participation and competition structures throughout NSW and the Australian Capital Territory (ACT) with a vested interest in promoting participation and competitive swimming at various representative levels (e.g., club, district, region, state, and national).
